# Synchronous Occurrence of Colloid Cyst With Intracranial Ossifying Fibromyxoid Tumor Masquerading as Meningioma

**DOI:** 10.7759/cureus.10662

**Published:** 2020-09-26

**Authors:** Sanjay Dhawan, Tusharindra Lal, P.N. Pandey, Ravindra Saran, Anutosh Singh

**Affiliations:** 1 Surgery, University of Minnesota, Minneapolis, USA; 2 Surgery, Sri Ramachandra Institute of Higher Education and Research, Chennai, IND; 3 Neurosurgery, Maulana Azad Medical College/Lok Nayak Hospital, New Delhi, IND; 4 Neuro-Pathology, Gobind Ballabh Pant Institute of Postgraduate Medical Education and Research/Maulana Azad Medical College, New Delhi, IND

**Keywords:** intracranial, ossifying fibromyxoid, meningioma, neurosurgery, radiology, brain tumor

## Abstract

Ossifying fibromyxoid tumor (OFMT) is a rare fibro-osseous neoplasm. We present a case highlighting the occurrence of an intracranial OFMT masquerading as meningioma on imaging in a 46-year-old gentleman. Brain imaging revealed an extra-axial calcified lesion along the left cerebellar convexity appearing hypointense on T1- and T2-weighted MRI sequences with no post-contrast enhancement, suggestive of a meningioma. An intraventricular colloid cyst was also noted. The lesion, which was presumed to be meningioma, and the colloid cyst were resected in two subsequent operative settings. Histopathological examination of the calcified lesion confirmed the findings of an OFMT. This report aims to inform the physician about intracranial OFMT mimicking meningioma on imaging. In addition, since multiple brain tumors are not very common, the surgeon should always have a suspicion should there be any heterogeneous and peculiar radiological and histopathological characteristics.

## Introduction

Ossifying fibromyxoid tumor (OFMT) was first described by Enzinger et al. in 1989 [1]. It is a rare fibro-osseous neoplasm displaying an uncertain histogenesis and an uncertain line of differentiation [1].

OFMTs are most commonly found in the subcutaneous tissues of extremities or trunk [2]. The head and neck is an uncommon anatomical location for OFMT. To the best of our knowledge, intracranial OFMTs not originating from the base of the skull or sinuses have not been previously reported in the literature. We describe a case of intracranial OFMT located in the left cerebellar convexity that closely mimicked a meningioma on imaging in an immunocompetent male who presented with an extra-axial calcified lesion. The patient was also found to have an intraventricular colloid cyst. It is also worthwhile to note that the evidence of simultaneously occurring primary brain tumors in patients who do not have genetic disorders, such as neurofibromatosis or tuberous sclerosis, or have no prior history of cranial radiotherapy remain poorly established in the literature. Although a handful of cases reporting the simultaneous occurrence of intraventricular lesions with gliomas have been published in the literature, this study is perhaps the first of its kind to also report a synchronous occurrence of an intracranial OFMT with an intraventricular colloid cyst.

The PubMed database was thoroughly reviewed for articles reporting intracranial OFMT on June 22, 2020. The keywords used in our search were “intracranial ossifying fibroma” and “intracranial fibromyxoid tumor” [All Fields].

## Case presentation

A 46-year-old gentleman presented to the neurosurgery outpatient clinic with complaints of headache and vomiting of one-year duration. There was no family history of any age-related sporadic or familial neoplastic illness. A thorough neurological and physical examination was performed, and no evidence of neurocutaneous disorders such as café au lait spots, Lisch nodule, or adenoma sebaceum was found. No intraocular lesions were noted. The patient was fully oriented with no neurological deficit.

**Figure 1:**
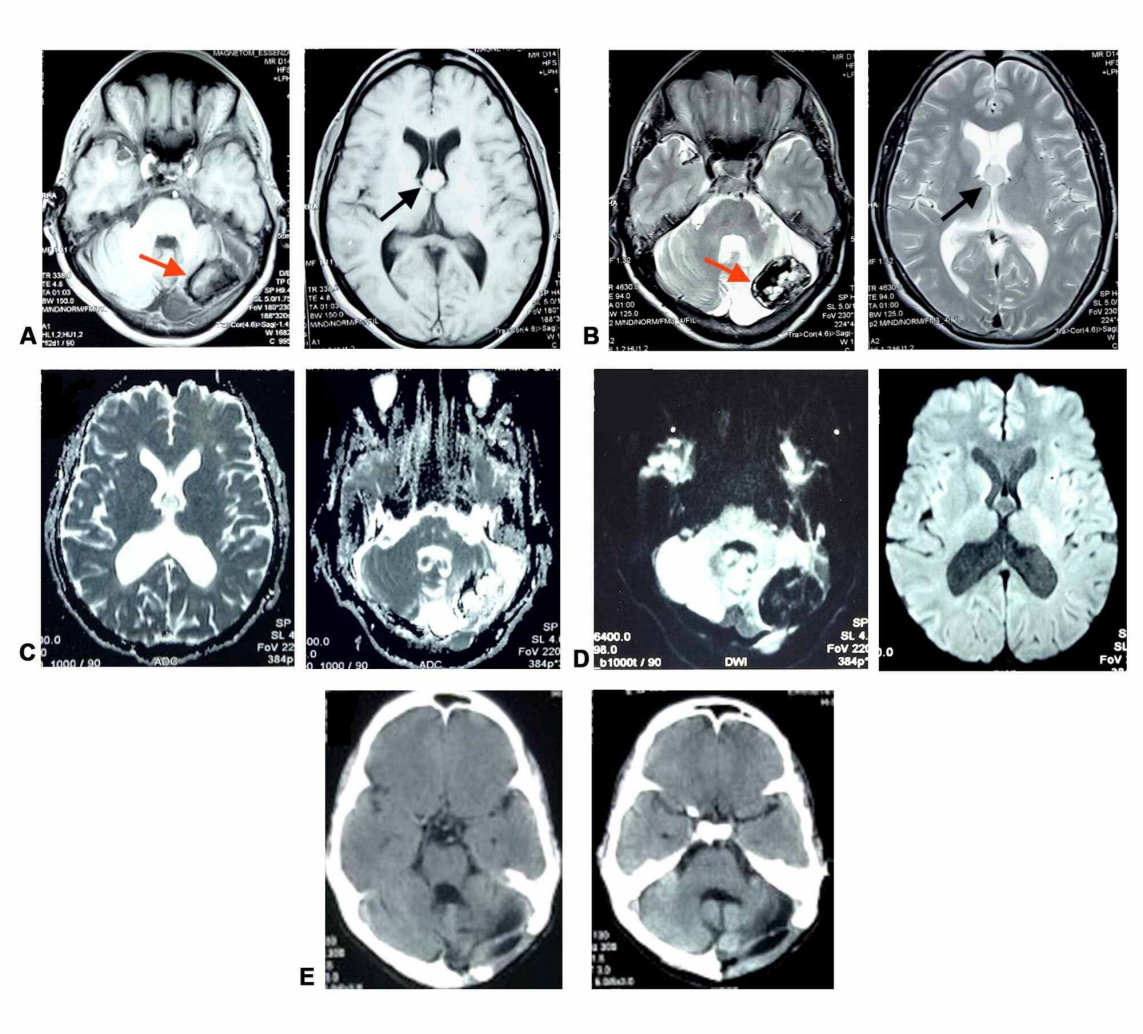
Well-defined extra-axial heterogeneous non-enhancing lesion seen in left cerebellar convexity with peripheral T1 and T2 hypointense rim and internal components showing mixed T2 hypo- and hyperintensity (red arrow). Left cerebellar atrophy is also seen. Another well-defined mildly T1 hyperintense and T2 isointense lesion is seen at the foramen of Monro, suggestive of colloid cyst (black arrow) (Figures A and B). Figures C and D demonstrate the DWI and the ADC sequences. (E) Post-operative CT scan following left sub-occipital craniectomy, showing complete excision of fibromyxoid cerebellar lesion. The hyperdense colloid cyst lesion can be seen. DWI, diffusion-weighted images; ADC, apparent diffusion coefficient; CT, computed tomography

Magnetic resonance imaging (MRI) of the brain revealed an extra-axial calcified lesion along the left cerebellar convexity appearing hypointense on T1-weighted (TIW) and T2-weighted (T2W) sequences, with no post-contrast enhancement and showing blooming on SWI (susceptibility-weighted imaging), suggestive of a meningioma (Figure 1). The scan also described another well-defined lesion near the foramen of Monro, appearing isointense to hyperintense on T1W, isointense to hypointense on T2W with no post-contrast enhancement with signal suppression on FLAIR (fluid-attenuated inversion recovery) and restricted diffusion within the lesion, compatible with a colloid cyst (Figure 1). MRI was the only pre-operative imaging performed.

Operation and post-operative course

**Figure 2:**
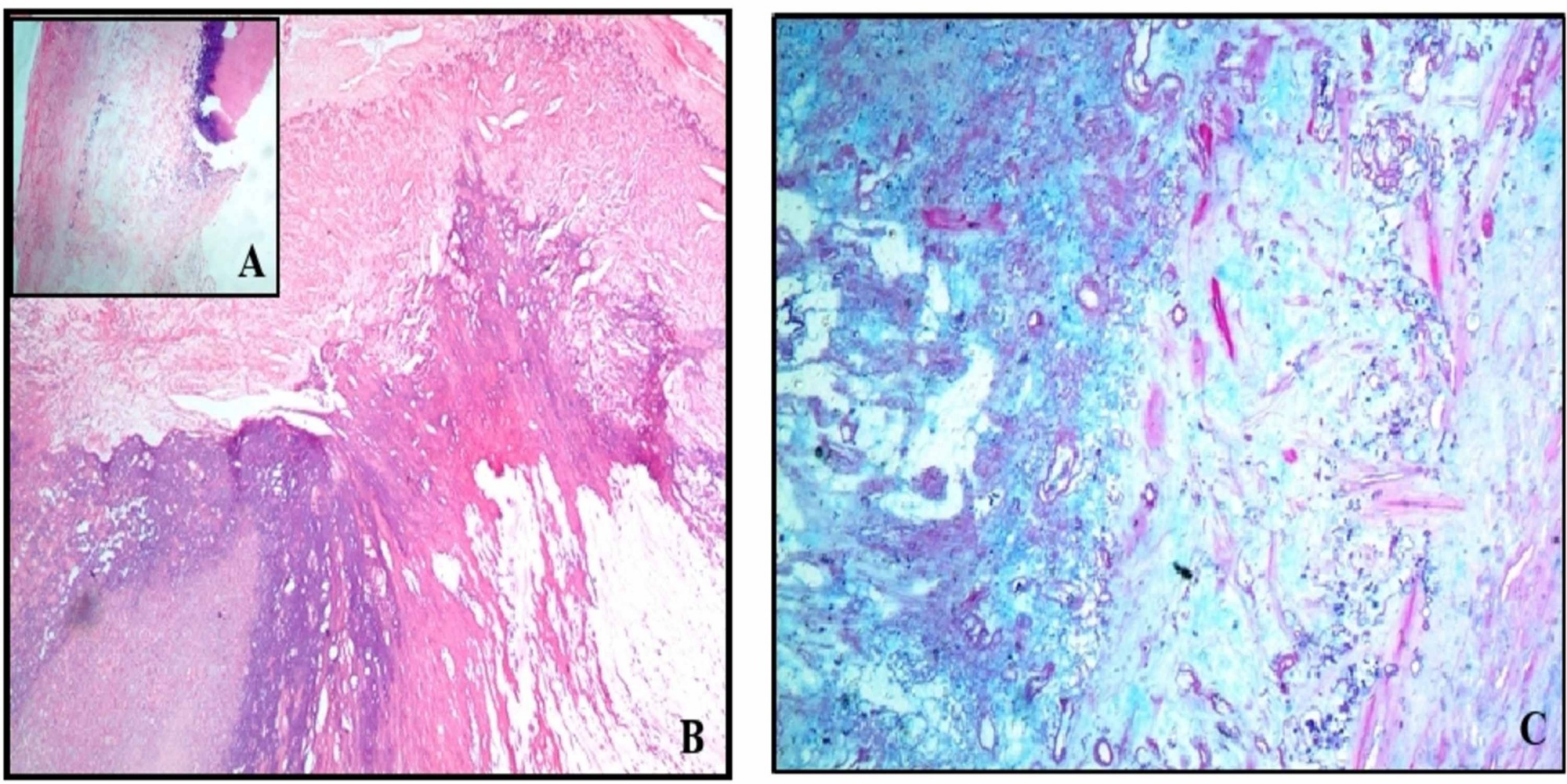
A section stained using hematoxylin and eosin reveals thickened dura mater with adjacent reactive gliosis and foci of dystrophic calcification, 10x (A), 20x (B). Periodic acid-Schiff-diastase/Alcian blue staining (20x) shows intervening pools of mucin (C).

The left cerebellar posterior fossa lesion was excised through a left sub-occipital craniectomy approach. Macroscopically, the tumor was tan, hard with a broad-based attachment. The post-operative course was uneventful. Post-operative cranial computed tomography (CT) revealed complete excision with a thin extra-axial bleed (~6 mm), with a colloid cyst in the third ventricle (Figure 1). On histopathological examination (HPE), the hematoxylin and eosin section revealed thickened dura mater with adjacent reactive gliosis and foci of dystrophic calcification and the periodic acid-Schiff-diastase (PAS-diastase)/Alcian blue staining demonstrated intervening pools of mucin, suggestive of an OFMT (Figure 2).

The patient was readmitted after eight weeks for the excision of the colloid cyst, which was performed through a right frontoparietal craniotomy through an anterior transcallosal approach. Grossly, the lesion was whitish, dome-shaped, partially obliterating the foramen of Monro, containing viscous fluid. Simple columnar epithelium lining fibrous wall with low cellularity on histopathology confirmed the lesion as a colloid cyst. The post-operative course was uneventful.

The patient has been following with us for the last two years and is doing fine, with no post-operative neurological deficit.

## Discussion

Enzinger et al. in a study on 59 cases described OFMT for the first time as a rare deep soft tissue tumor of uncertain differentiation predominantly occurring in the adult male population (male-to-female ratio of 1.5:1). Majority of the cases involved the lower extremities [1]. Relevant clinical studies that were available in English language are described in Table 1 [3-17].

**Table 1:** Studies that discuss intracranial ossifying fibromas. CT, computed tomography; MRI, magnetic resonance imaging; T1W, T1-weighted; -, not reported

Author, year	Country	Area involved	Imaging	Histopathology
Scott et al., 1971 [3]	USA	Orbits, paranasal sinuses, right maxillary antrum with intracranial extension	Ossifying fibroma involving the mid-anterior fossa bilaterally, and extending posteriorly to the clinoid processes	-
Tomita et al., 1981 [4]	USA	Maxillary sinus with intracranial extension	CT: involvement of nasal cavity with base of the skull and cribriform plate	Highly cellular fibroblastic proliferation with foci of ossification and calcification. Herringbone pattern arrangement of fibroblastic spindle cell proliferation
Ito et al., 1984 [5]	Japan	Right frontoethmoid sinus	CT: dense bony tumor with sharp demarcation, eggshell appearance extending into the right frontoethmoid sinus, the right orbit, and intracranial cavity	Small calcified spicules interspersed freely in dense fibrous stroma without mitosis. No psammoma bodies.
Blitzer et al., 1989 [6]	USA	Fibro-osseous lesions of the frontal and ethmoidal sinuses	CT: ossified lesion of the paranasal sinuses	-
Wenig et al., 1995 [7]	USA	Sinonasal tract	Osseous, soft tissue mass, ± bone erosion and intracranial extension (anterior cranial fossa)	Psammomatoid bodies admixed with myxomatous material and scattered giant cells
Ruggieri et al., 1996 [8]	Italy	Frontoparietotemporal ossifying fibroma with intracranial growth	CT: thickening of both the right sphenoidal wing and the right temporal table T1W MRI: parietal bone lesion displacing the frontoparietal brain lobes	Lamellar bone with cellular fibrous stroma
Noudel et al., 2009 [9]	France	Right paranasal sinuses	CT: sinonasal mass protruding into the right globe laterally. T1W MRI: well-circumscribed soft tissue mass with homogenous enhancement filling the right nasal cavity, ethmoid sinus with slight extension through the anterior cranial fossa	Densely fibrous stroma composed of fusiform cells with storiform appearance with round basophilic calcifications
Kansal et al., 2010 [10]	India	Petromastoid bone with intracranial extension	CT: expansile lesion of the right petromastoid bone MRI: contrast-enhancing mass involving the right petromastoid bone with extradural extension compressing the cerebellar hemisphere	Fibroblastic proliferation with cementum and bony trabeculae
Sarode et al., 2011 [11]	India	Left mandible	CT: multilocular expansile lesion of the left mandible with heterogeneous attenuation	Densely cellular fibrous stroma interspersed with psammoma bodies
Rowland et al., 2013 [12]	USA	Skull base	CT: sphenoidal bone ossifying fibroma filling the sphenoidal sinus and extending intracranially. Left abducens nerve involvement on MRI	Cystic transformation with active processes
Lee et al., 2014 [13]	Republic of Korea	Temporal bone	CT case I: 2.9 cm calcified mass in the temporal bone; case II: 5.5 cm enhancing mass with internal cartilaginous matrix in the temporal bone	Osseous islands scattered throughout the bland fibrous stroma
Al-Sharhan et al., 2016 [14]	Saudi Arabia	Ethmoid sinus	CT: well-defined expansile lesion of the mid-ethmoid and frontal sinus with extensive bone remodelling and thinning of the adjacent lamina papyracea and superior orbital roof, with internal heterogeneous soft tissue component. MRI: concentric soft tissue and multiple fluid levels of high signal intensity on T1 and T2 sequences with mild enhancement post-contrast administration	Numerous small ossicles or psammomatoid bodies embedded in the cellular fibrous stroma with cysts lined by fibroblasts and histiocytes
Ghosal et al., 2016 [15]	India	Left maxillo-ethmoid sinus lesion	CT: calcifications within the lesion; T1W MRI: lobulated enhancing mass lesion in left frontal and ethmoid sinuses with intracranial extension into the crista galli and anterior interhemispheric fissure	Oval to spindle cells dispersed in a myxoid extracellular matrix and arranged in focal lobulated architecture
Jiang et al., 2018 [16]	USA	Squamous suture of the temporal bone	CT: Bullough’s bump of the left temporal bone	Oval osseous islands dispersed throughout a bland fibrous stroma. The pathological diagnosis was "Bullough's bump", a rare, benign fibro-osseous neoplasm
Mukherjee et al., 2019 [17]	India	Orbit	CT: lobulated expansile fibro-osseous lesion involving the greater wing of the sphenoid and orbital roof without intracranial extension	Fibroblast rich stroma with bony trabeculae; osteoblastic rimming without any mitotic activity

The brain imaging in our case revealed an extra-axial calcified lesion along the left cerebellar convexity with features suggestive of a calcified meningioma. HPE revealed thickened dura mater and fibro-collagenous tissue showing foci of dystrophic calcification with low cellularity and mitotic rate < 2/50 high powered fields (HPF), suggestive of a “typical OFMT”. The HPE findings were consistent with the Folpe and Weiss classification of OFMT, which described “typical OFMT” as those showing low nuclear grade and low cellularity and mitotic rate < 2/50 HPF [2]. In contrast, malignant OFMT shows a high nuclear grade or high cellularity and mitotic activity > 2/50 HPF [2].

OFMT is a much rarer entity as compared to meningiomas, which account for 14%-19% of the primary intracranial neoplasms [18]. We would like to highlight that an OFMT lesion on imaging could masquerade as a calcified meningioma, like in this case.

Although most malignant OFMT may be recognized histologically, a small number of otherwise typical OFMT may behave in a clinically malignant fashion, supporting their reclassification as tumors of intermediate malignancy [2]. Recognition of malignant OFMT should assist in the clinical management of patients with this rare soft tissue neoplasm. Immunohistochemistry analysis of the ossifying fibromyxoid lesion revealed positivity for S-100 (polyclonal, 1:800, Dako, Glostrup, Denmark), suggesting Schwannian differentiation. This conforms to the concept of neuronal differentiation of OFMT as laid by Folpe and Weiss [2]. Immunohistochemical staining for markers such as NSE and CD57 would be helpful in the meningioma diagnosis of such lesions, which can help us differentiate these lesions better from OFMTs.

To the best of our knowledge, this case is also the first documentation depicting the synchronous occurrence of an OFMT with an intraventricular colloid cyst. Different hypotheses exist, which explain the coexistence of multiple primary intracranial tumors of different histogenesis in different compartments of the brain in patients without neurocutaneous disorders or cranial radiotherapy. Gelabert et al. reported the simultaneous occurrence of a frontal lobe astrocytoma and a colloid cyst of the third ventricle, both derived from displaced primitive neuroectodermal cells, suggesting the role of a single oncogenic factor in producing different tumors in the same individual [19]. Another hypothesis suggested by Karami et al. explains that this phenomenon of simultaneous occurrence of histologically different tumors in different or same areas of the brain could be purely coincidental [20].

There is no defined treatment strategy regarding which lesion should be treated first in patients with simultaneous brain tumors. We operated the OFMT first followed by the excision of the colloid cyst. It can be said that it is the surgeon’s preference in corroboration with the patient profile as to which lesion should be dealt with first.

## Conclusions

OFMTs are believed to commonly arise from the skull base locations or paranasal sinuses. This case reports the presence intracranial OFMT arising from the left cerebellar convexity. We believe this case would help in informing the physicians regarding the management of extra-axial calcified lesions where an OFMT could masquerade as a calcified meningioma, as reported in this case. This is also the first case to report synchronous occurrence of intracranial fibromyxoid tumor with a colloid cyst. Since synchronous brain tumors are not very common, the surgeon should always have a suspicion should there be any heterogeneous and peculiar radiological and histopathological characteristics.
